# Phosducin-like 3 is a novel prognostic and onco-immunological biomarker in glioma: A multi-omics analysis with experimental verification

**DOI:** 10.3389/fimmu.2023.1128151

**Published:** 2023-03-15

**Authors:** Zesheng Peng, Jiajing Wang, Shiao Tong, Yuxi Wu, Dongye Yi, Wei Xiang

**Affiliations:** ^1^ Department of Neurosurgery, Union Hospital, Tongji Medical College, Huazhong University of Science and Technology, Wuhan, Hubei, China; ^2^ Department of Neurosurgery, Renmin Hospital of Wuhan University, Wuhan, Hubei, China

**Keywords:** glioma, PDCL3, prognostic biomarker, immune landscape, tumor microenvironment

## Abstract

Malignant glioma is the most frequent primary tumor of the central nervous system. PDCL3 is a member of the phosducin-like protein family, and its imbalance has been shown to be associated with several human diseases. However, the underlying role of PDCL3 in human malignant cancers, especially in malignant gliomas, is unclear. In this study, we combined public database analysis and experimental verification to explore the differential expression, prognostic value and potential functions and mechanisms of PDCL3. The results revealed that PDCL3 is upregulated in multiple cancers and acts as a potential prognostic biomarker of glioma. Mechanistically, PDCL3 expression is associated with epigenetic modifications and genetic mutations. PDCL3 may directly interact with the chaperonin-containing TCP1 complex, regulating cell malignancy, cell communication and the extracellular matrix. More importantly, the association of PDCL3 with the infiltration of immune cells, immunomodulatory genes, immune checkpoints, cancer stemness and angiogenesis suggested that PDCL3 may regulate the glioma immune landscape. Furthermore, PDCL3 interference also decreased the proliferation, invasion and migration of glioma cells. In conclusion, PDCL3 is a novel oncogene and can be adopted as a biomarker with value in assisting clinical diagnosis, predicting patient outcomes and assessing the immune landscape of the tumor microenvironment in glioma.

## Introduction

Malignant glioma is the most frequent primary tumor of the central nervous system in adults (1). Although modern medicine has made great achievements in microsurgery, molecular diagnosis and treatment, precision radiotherapy and other fields, the prognosis of patients with malignant glioma is still unsatisfactory (2). Patients with glioblastoma multiforme (GBM, WHO IV) have a median survival of only 14 months from diagnosis (3). Although the prognosis of lower-grade glioma (LGG, WHO II - III) is relatively optimistic, almost 70% of LGG will progress to secondary glioblastoma in a few years (4). Recent advances in the molecular pathology of gliomas have greatly aided in the accurate diagnosis and therapeutic strategy of glioma (5). Molecular biomarkers, represented by isocitrate dehydrogenase (IDH) mutation, 1p19q codeletion and O 6-methylguanine-DNA methyltransferase promoter (MGMTp) methylation, were updated to guidelines and partly benefited patients (6); however, these most widely utilized biomarkers cannot yet adequately reflect individual heterogeneity. In fact, the large amount of data generated from The Cancer Genome Atlas Project (TCGA) has confirmed the existence of subtypes with distinct pathological molecular events and therapeutic responses in glioma (7). This heterogeneity emphasizes the opinion that no single target is likely to control the malignant progression of all gliomas (8). Therefore, more studies are urgently needed to elucidate the potential biological mechanisms, establish novel drug targets, and identify effective biomarkers for malignant gliomas.

Phosducin-like 3 (PDCL3) is also known as Phosducin-like Protein 2A (PhLP2A) (9) or Viral IAP associated factor (VIAF) (10). This gene encodes a member of the phosducin-like protein family, which all share an N-terminal helical domain, a central thioredoxin-like domain, and a charged carboxyl terminus (11). PDCL3 was originally identified as a protein that is involved in G protein signaling by binding with the Gβγ dimer and was thought to promote G protein function by interacting with chaperone proteins containing TCP-1 (CCT) to assist the correct folding of proteins or by directly regulating Gβγ dimer assembly (9). In addition, an earlier study also found that PDCL3 may act as a regulator of apoptosis in mammalian cells, but the significance and mechanism of this function is unclear (10). Furthermore, recent studies have found that PDCL3 may act as a chaperone protein involved in the regulation of VEGFR-2 expression and function (12). This phenomenon is regulated by both hypoxia induction and N-terminal methionine acetylation of PDCL3 itself (13). Gene mutations or aberrant expression of PDCL3 have been shown to be associated with Alzheimer’s disease (14), fetal macrovesicular microcolon (15), and severe dengue infections (16). In addition, another member of the phosducin-like protein family, phosducin-like protein 3 (PhLP3), also known as thioredoxin domain-containing 9 (TXNDC9), was proven to be an oncogene that promotes the malignant progression of many types of cancers, including prostate cancer (17), hepatocellular carcinoma (17), colorectal adenocarcinoma (18), gastric cancer (19) and malignant glioma (20). However, the pathological role of PDCL3 in human cancers has not been investigated, and a comprehensive study on PDCL3 expression, prognostic value, and the underlying mechanisms across cancers, especially in malignant glioma, needs to be performed.

Here, by using bioinformatics analysis and experimental validation, we have provided evidence showing that PDCL3 is upregulated in multiple cancers and suggests a poor prognosis in patients. The abnormal expression of PDCL3 is not only closely related to the clinical characteristics, prognosis and tumor immune microenvironment of glioma but is also involved in promoting the malignant progression of glioma cells. The details of this research are reported as follows.

## Materials and methods

### Data sources

Gene expression data were obtained from the TCGA database (https://portal.gdc.cancer.gov/), the Chinese Glioma Genome Atlas (CGGA; http://www.cgga.org.cn/), the Genotype-Tissue Expression database (GTEx; https://gtexportal.org/) and the Cancer Cell Line Encyclopedia database (CCLE; https://portals.broadinstitute.org/ccle/about). All RNA-seq data were obtained in the format of fragments per kilobase of exon model per million mapped reads (FPKM) or transcripts per kilobase of exon model per million mapped reads (TPM) normalized. The corresponding clinical information of patients was also obtained from the above databases.

### Bioinformatics analysis

The Sangerbox tool (http://www.sangerbox.com/tool), a free data analysis platform, was used to validate the pan-cancer expression of PDCL3 and explore the correlation of PDCL3 expression with the expression of RNA modification genes, DNA methyltransferases, immunomodulatory genes and immune checkpoint genes, as well as single nucleotide variation frequency, cancer stemness and multiple immune infiltration scores, by using Pearson’s method. The Tumor IMmune Estimation Resource (TIMER; https://cistrome.shinyapps.io/timer/) and the Human Protein Atlas (HPA; https://www.proteinatlas.org) were also applied to validate PDCL3 expression in pan-cancer tissues and cancer cell lines. The log-rank test was used to examine Kaplan-Meier (KM) survival analysis comparing patient outcomes (overall survival and progression-free survival) between the high and low PDCL3 groups. ROC curve analysis was utilized to quantify the diagnostic efficacy and prognostic power of PDCL3. Logistic regression was performed to assess the correlation between PDCL3 expression and clinicopathological characteristics. For functional analysis of the PDCL3 gene in glioma, the TCGA glioma dataset was divided into high and low PDCL3 groups according to its expression. The DEGs between the two groups were identified (|log2FC|>1 and adjusted p<0.05) and functionally annotated by Gene Ontology (GO) and Kyoto Encyclopedia of Genes and Genomes (KEGG) pathway analyses *via* the R packages “clusterProfiler”, “org.Hs.e.g.db” and “enrichplot”. Gene set enrichment analysis (GSEA) was also carried out for further mechanistic analysis based on the “c2.cp.v7.2.symbols.gmt” gene sets by using the JAVA program. For functional analysis of the PDCL3 protein, the free platform LinkOmics (http://www.linkedomics.org/login.php) was applied to online analyze the proteome dataset of the GBM cohort in the Clinical Proteomic Tumor Analysis Consortium (CPTAC). GeneMANIA (http://www.genemania.org) was used to construct the protein interaction network of PDCL3. Furthermore, two free single-cell sequencing data platforms, CancerSEA (http://biocc.hrbmu.edu.cn/CancerSEA/home.jsp) and TISCH (http://tisch.comp-genomics.org/home/), were applied to validate the expression and function of PDCL3 at the single-cell level.

### Establishment and evaluation of a nomogram

Univariate and multivariate Cox regression analyses were used to determine the independent prognostic value of PDCL3. Subsequently, a nomogram based on independent prognostic factors in the TCGA cohort was established by using the R package “rms”. Calibration curves at 1, 3, and 5 years were plotted for graphical evaluation. The concordance index (C-index) and the ROC curve were used to assess the predictive accuracy of the nomogram.

### Glioma tissue samples and tissue microarray

A total of 94 human glioma tissues and corresponding clinical information were obtained from the Department of Neurosurgery of Wuhan Union Hospital from July 2015 to July 2021. The clinicopathological characteristics of the patients are summarized in **Additional File 1**. Fresh tumor tissues were resected and immediately preserved in liquid nitrogen or 10% formalin for subsequent testing. Glioma tissue procurement and use were performed with written patient informed consent and approved by the Institutional Ethics Committee of Tongji Medical College, Huazhong University of Science and Technology. The glioma tissue microarray and its corresponding clinical data were presented by Professor Junhui Liu from Renmin Hospital of Wuhan University. The fabrication and use of this tissue microarray was approved by the Ethics Committee of Renmin Hospital of Wuhan University, and all patients signed informed consent forms. The clinicopathological characteristics of the patients are summarized in **Additional File 2**.

### Cell culture and transfection

The human glioma cell lines U251 and U87-MG were purchased from the Cell Bank Type Culture Collection of the Chinese Academy of Sciences (Shanghai, China) and identified by Procell Life Science & Technology Co., Ltd. (Wuhan, China). Cells were cultured in Dulbecco’s modified Eagle’s medium (DMEM, Cytiva, USA) with 10% fetal bovine serum (Gibco, Thermo Fisher Scientific, USA), penicillin (100 U/mL) and streptomycin (100 µg/mL) at 37°C under a humidified atmosphere of 5% CO2. Commercialized PDCL3 knockdown lentiviruses and their negative controls were purchased from GeneChem Co., Ltd. (Shanghai, China). Lentivirus transfection was performed according to the manufacturers’ protocol. The screening and purification of infected cells was performed by culture in medium containing puromycin, and PDCL3 knockdown efficiency was detected using qRT-PCR and Western blotting. Full-length human PDCL3 (NM_024065.5) cDNA was subcloned into a pCMV-myc vector to generate the myc-PDCL3 construct. The plasmid transfection was performed using Lipofectamine 3000 (Thermo Fisher Scientific, USA) according to the manufacturer’s instructions.

### Quantitative real-time polymerase chain reaction (qRT-PCR)

Total RNA of each glioma tissue or cell sample was extracted using TRIzol reagent (Invitrogen). According to the manufacturer’s instructions, cDNA was synthesized by reverse transcription using a reverse transcription kit (Takara RR036A). qRT-PCR analysis was further performed on a LightCycler 480 Real-Time PCR system using TB Green^®^ Premix Ex Taq™ II (Takara RR820A). GAPDH was used for normalization, and the comparative Ct method (ΔΔCt) was used to evaluate mRNA expression. The primer sequences are listed in **Additional File 3**.

### Western blot

U251 and U87-MG cell lysates were prepared by sonicating cells briefly in a modified RIPA buffer (Biosharp, China) with proteinase and phosphatase inhibitors. The protein concentration was measured using a BCA protein assay kit (Beyotime Biotechnology, China) according to the instructions. Protein samples were separated by 10% SDS-PAGE and transferred to a PVDF membrane (Millipore, USA), blocked in 5% nonfat milk at room temperature for 1 h and immunoblotted with primary antibodies at 4°C overnight. After washing with 1× TBST three times, the membranes were incubated with the appropriate horseradish peroxidase-conjugated secondary antibody for 2 h. The results were visualized with ECL reagent and developed. All antibodies used in the experiments are listed in **Additional File 4**.

### RNA sequencing and immunoprecipitation-mass spectrometry (IP-MS)

U251 cells infected with PDCL3-3 knockdown lentivirus and its control lentivirus were collected, and their RNA was extracted for transcriptome sequencing (biological replicates were performed three times). Subsequently, the DEGs were screened out (|log2FC|>0.5 and q-value<0.05) and are shown with a heatmap. GO terms and KEGG analysis were used to annotate and explore potential functional mechanisms. For IP-MS, U251 cells infected with myc-PDCL3 were lysed in IP buffer containing 1% NP-40, 50 mM NaF, 2 mM Na3VO4, 4 mM Na pyrophosphate and protease inhibitors. A total of 3 μg of mouse anti-myc monoclonal antibody or IgG was added to the cell lysates, and 4 hours later, the samples were incubated with 30 μL Protein A/G (Beyotime, China) at 4°C overnight. The precipitates were washed 5 times with cold PBS and boiled for 5 min in 40 μL of 2x loading buffer (Biosharp, China), followed by SDS-PAGE and silver staining. Mass spectrometry and analysis were performed to identify the immunoprecipitation-enriched proteins.

### Immunofluorescence and Immunohistochemistry

For immunofluorescence (IF), tissue sections were deparaffinized, hydrated and subjected to antigen retrieval in 10 mM sodium citrate (pH 6.0). Then, the sections were incubated with the primary antibody overnight, followed by CY3-labeled secondary antibody. After antigen retrieval, the sections were incubated with the second primary antibody overnight, followed by FITC-labeled secondary antibody. After antigen retrieval again, the sections were incubated with the third primary antibody overnight, followed by CY5-labeled secondary antibody. Finally, the nuclei were stained with DAPI (Servicebio, Wuhan). For immunohistochemistry (IHC), formalin-fixed paraffin tissue and tissue microarrays were used. Briefly, the sections were deparaffinized, hydrated and subjected to antigen retrieval in 10 mM sodium citrate. Endogenous peroxidase was blocked in 3% H2O2 for 10 min. The sections were incubated in primary antibodies overnight, followed by HRP-labeled secondary antibody. Signals were detected using diaminobenzidine (DAB) staining, and the sections were counterstained with hematoxylin. The IF and IHC images were obtained using an inverted phase contrast microscope (Olympus IX73), and ImageJ software was used to quantify the expression of protein, which was presented as the ratio of average optical density (AOD): AOD = integral optical density (IOD)/positive area.

### Cell proliferation, invasion and migration assays

5-Ethynyl-2’-deoxyuridine (EdU) assay was utilized to monitor the proliferation of transfected cells. The treated U251 and U87-MG cells were incubated with 10 µM EdU for 4 hours after adherence to a 96-well plate and then fixed, permeabilized, and stained with both Alexa Fluor 594 reaction cocktail for EdU and DAPI for cell nuclei. Finally, the samples were imaged under a fluorescence microscope. Transwell assays were used to measure cell invasion. Before the Transwell assay, cells were starved in serum-free medium for 24 h to remove the effect of proliferation on the experimental results. Appropriate numbers of glioma cells were added to Matrigel-coated upper Transwell chambers (Corning, USA) for the invasion assay. The lower chamber was filled with 500 µl of DMEM containing 10% FBS. After incubation at 37°C for 24 h, cells in the upper chamber were fixed with 4% paraformaldehyde for 15 min, stained with 0.1% crystal violet for 15 min and counted under an inverted microscope. A wound healing assay was used to test the cell migration ability. In brief, cells were seeded in a 6-well plate and cultured for a certain time to reach approximately 90% confluence. A sterile pipette tip was used to scratch a linear wound, and serum-free DMEM was added for further culturing for 48 h. Wound healing images were captured using an inverted microscope, and ImageJ software was used to analyze the relative area of wound closure.

### Statistical analysis

The downloaded data were organized using Excel software. Most data analysis and visualization were performed mainly by R software (v3.6.1), and the rest were performed with GraphPad Prism (v9.0.0). The results are representative of three independent experiments and presented as the mean ± SD. Student’s t test or one-way ANOVA (followed by Bonferroni *post hoc* tests) was utilized to compare continuous variables between two groups or more than two groups. A nonparametric test was used to compare the expression of the PDCL3 gene or protein between glioma tissues. The log-rank test was used for survival analysis, and the Pearson test was used for correlation analysis. All statistical tests were bilateral, and a P value < 0.05 was considered statistically significant.

## Results

### PDCL3 is upregulated in multiple cancers and predicts poor prognosis

**Figure 1:**
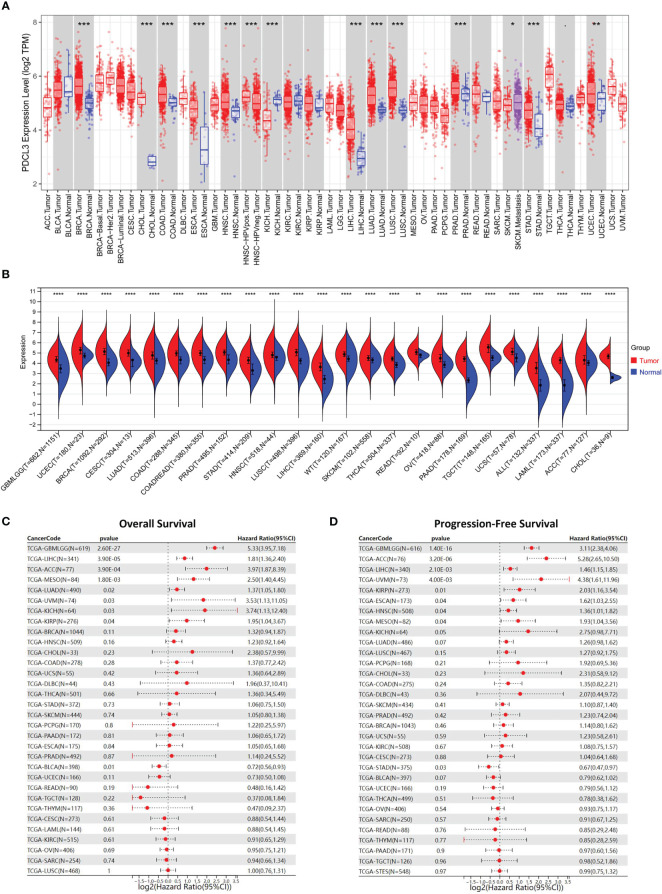
PDCL3 is upregulated in multiple cancers and predicts poor prognosis. **(A)** The expression of PDCL3 across cancers from the TIMER database. **(B)** The expression of PDCL3 across cancers based on TCGA and GTEx databases. **(C)** Forest plots showing the correlation between PDCL3 expression and overall survival across cancers. **(D)** Forest plots showing the correlation between PDCL3 expression and progression-free survival across cancers. *p<0.05, **p<0.01, ***p<0.001, and ****p<0.0001.

We first analyzed the expression profiles of PDCL3 in various human cancer tissues and cell lines. As shown in **Figure 1A**, PDCL3 is significantly upregulated in 12 types of cancers, including breast invasive carcinoma (BRCA), cholangiocarcinoma (CHOL), colon adenocarcinoma (COAD), esophageal carcinoma (ESCA), head and neck squamous cell carcinoma (HNSC), kidney chromophobe (KICH), liver hepatocellular carcinoma (LIHC), lung adenocarcinoma (LUAD), lung squamous cell carcinoma (LUSC), prostate adenocarcinoma (PRAD), stomach adenocarcinoma (STAD) and uterine corpus endometrial carcinoma (UCEC). In addition, among multiple human cancer cell lines, PDCL3 expression in brain tumors was also relatively high (**Figures S1A, B**). Furthermore, due to the lack of adequate control samples in TCGA, normal tissue data from the GTEx database were integrated to analyze the expression differences of PDCL3 in various tumors. The results showed that PDCL3 expression was upregulated in 22 types of tumor tissues compared with normal controls (**Figure 1B**), including GBMLGG, UCEC, BRCA, cervical squamous cell carcinoma and endocervical adenocarcinoma (CESC), LUAD, COAD, PRAD, STAD, HNSC, LUSC, LIHC, skin cutaneous melanoma (SKCM), thyroid carcinoma (THCA), rectum adenocarcinoma (READ), ovarian serous cystadenocarcinoma (OV), pancreatic adenocarcinoma (PAAD), testicular germ cell tumors (TGCT), uterine carcinosarcoma (UCS), acute lymphoblastic leukemia (ALL), acute myeloid leukemia (LAML), adrenocortical carcinoma (ACC) and CHOL. To explore the relationship of PDCL3 expression with patient outcomes, TCGA pan-cancer expression data and clinical information were integrated, and samples with no expression of PDCL3 or a follow-up time less than 30 days were excluded. Subsequently, the Cox proportional hazards regression model was established, and the log-rank test was used to obtain the prognostic significance. Finally, we observed that high PDCL3 expression was associated with poor overall survival (OS, **Figure 1C**) and progression-free survival (PFS, **Figure 1D**) in eight different types of cancer, with the most significant relationship being glioma.

### Upregulated PDCL3 is a novel prognostic biomarker in malignant glioma

**Figure 2:**
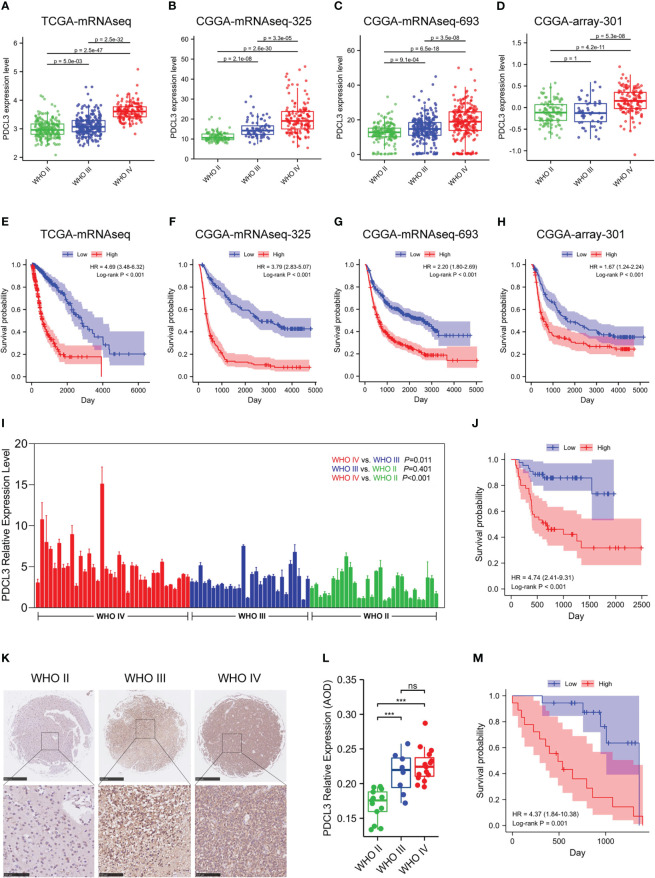
Upregulated PDCL3 is a prognostic biomarker in glioma. **(A–D)** Stratification analysis of PDCL3 expression in four different datasets. **(E–H)** Kaplan-Meier curves showing the OS of the PDCL3 high and low groups in four different datasets. **(I)** PDCL3 mRNA expression in the cohort composed of 94 glioma patients. **(J)** Kaplan-Meier curves of this 94-patient cohort. **(K)** Representative pictures of IHC staining in the glioma tissue microarray cohort (scale bars of upper = 500 µm, scale bars of lower = 100 µm). **(L)** PDCL3 protein expression in the glioma tissue microarray (***p<0.001 and ns, no significance). **(M)** Kaplan-Meier curves of this microarray cohort.

To further explore the expression and prognostic value of PDCL3 in malignant glioma, TCGA and three other cohorts (CGGA-mRNAseq-693, CGGA-mRNAseq-325 and CGGA-array-301) were used for stratification analysis and validation. The results reflected that PDCL3 expression was significantly associated with the pathological grade and prognosis of glioma patients. As the WHO grade of glioma samples increased continuously, the expression level of PDCL3 also showed a corresponding upregulated trend (**Figures 2A–D**). The ROC curves showed AUC of TCGA = 0.905, AUC of CGGA325 = 0.835, AUC of CGGA693 = 0.692 and AUC of CGGA301 = 0.761, which confirmed its potent accuracy in all cohorts (**Figure S2A–D**). The KM survival curve indicated that the OS of glioma patients in the low PDCL3 groups was significantly better than that in the high PDCL3 groups (**Figures 2E–H**). A satisfactory prediction performance of PDCL3 was confirmed by time-dependent ROC curves for 1-, 3-, and 5-year OS (**Figure S2E–H**). Moreover, we also examined the mRNA and protein expression levels of PDCL3 in our glioma tissue cohort and tissue microarray by qRT-PCR and IHC assays and performed survival analysis with the corresponding clinical data. In the cohort composed of 94 human glioma tissues, the mRNA expression of PDCL3 in WHO grade IV glioma was significantly higher than that in WHO grade II or WHO grade III glioma (**Figure 2I**), and patients with higher PDCL3 mRNA expression had poorer survival times (**Figure 2J**). In the glioma tissue microarray cohort, the protein expression of PDCL3 in WHO II grades was significantly lower than that in WHO III and WHO IV grades (**Figure 2L**), and patients with lower PDCL3 protein expression had a better prognosis (**Figure 2M**). Representative pictures of IHC staining are shown in **Figure 2K**.

### Construction and evaluation of the nomogram

**Figure 3:**
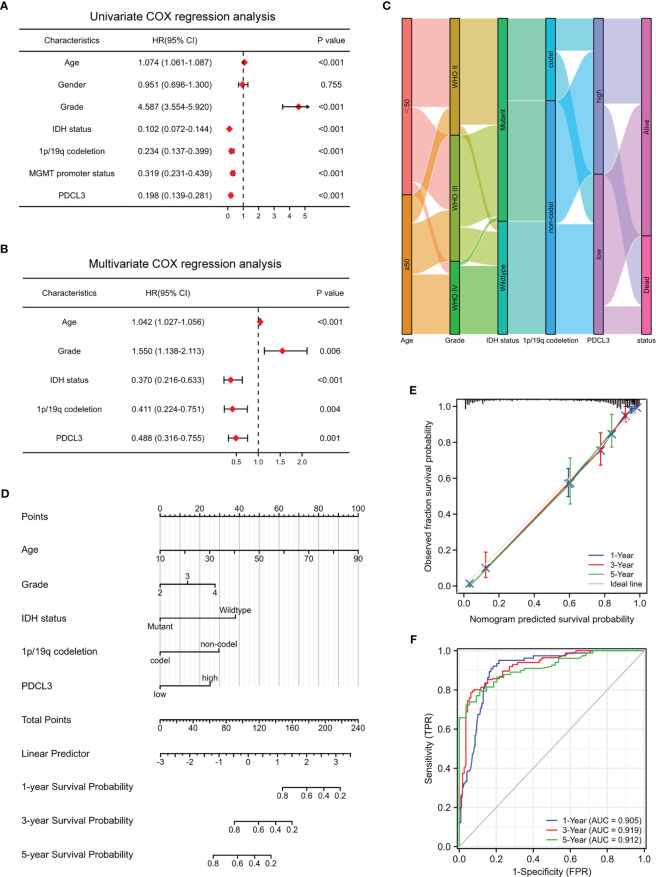
Construction and evaluation of the nomogram. **(A, B)** Univariate and multivariate Cox regression analyses in the TCGA cohort. **(C)** Sankey diagram showing the overall prognostic trend and living status of the inner relationship. **(D)** Nomogram based on age, WHO grade, IDH status, 1p/19q codeletion and PDCL3 expression. **(E)** Calibration curves showed the concordance between predicted and observed 1-, 3-, and 5-year OS. **(F)** ROC curve analyses of the nomogram in predicting 1-, 3-, and 5-year OS.

To assess the correlation between PDCL3 expression and each clinicopathological characteristic, logistic regression was performed. The results indicated that the categorical dependent variable PDCL3 expression was correlated with poor prognostic clinical parameters, including age, WHO grade, histological type, IDH status and 1p/19q codeletion (**Supplementary Table 5**). To further identify whether PDCL3 can be qualified as a prognostic predictor of glioma, we conducted univariate and multivariate Cox regression analyses in combination with common clinicopathological characteristics. The results reflected that PDCL3 not only revealed satisfactory prognostic efficiency, similar to age, tumor grade, IDH status, 1p19q codeletion and MGMT promoter unmethylated status (**Figure 3A**), but was also an independent predictor in the multivariate Cox regression analysis (**Figure 3B**). A Sankey diagram was used to visualize the overall prognostic trends of patients with high and low PDCL3 expression, as well as the relationships among the four independent factors (age, WHO grade, IDH status and 1p/19q codeletion) in living status (**Figure 3C**). These results further illustrated that PDCL3 could serve as a reliable and novel prognostic biomarker. We subsequently established a nomogram (**Figure 3D**) in the TCGA cohort. The C-index of this nomogram was 0.864, and the calibration plots revealed an excellent match between the actual and nomogram-predicted probability of 1-, 3-, and 5-year OS (**Figure 3E**). The ROC curves presented excellent sensitivity and specificity of the prognostic PDCL3 (1-year AUC = 0.905,3-year AUC = 0.919,5-year AUC = 0.912; **Figure 3F**). These results together confirmed that the nomogram had satisfactory prognostic efficiency for glioma, and it had the potential to be developed into a quantitative tool to predict the prognosis of glioma patients.

### PDCL3 expression is associated with epigenetic modifications and genetic mutations

Epigenetic modifications have the possibility of functional gene modification, which has been proven to play important roles in tumorigenesis in multiple cancers. DNA methylation and RNA methylation, including N1-methyladenosine (m1A), cytosine-5-methylation (m5C) and N6-methyladenosine (m6A), are the most common DNA and RNA modifications, so we explored their correlations with PDCL3 expression levels across cancers. As shown in **Figures S3A, B**, PDCL3 expression is closely related to the expression of 44 RNA modification genes (m1A [n=10], m5C [n=13], and m6A [n=21]) and 4 DNA methyltransferase genes (DNMT1, DNMT2, DNMT3A, and DNMT3B) in multiple tumors, which indicates that PDCL3 may mediate malignant progression by epigenetic modification directly or indirectly. To gain further insight into PDCL3-related mechanisms, we also analyzed the genetic mutation profile in the TCGA cohort. By organizing simple nucleotide variation (SNV) datasets, we found that F missense mutations (0.2%) were the main type in pan-cancers. **Figure S3C** displays the PDCL3 mutation distribution and types in its protein domains. Waterfall diagrams were used to present the top 15 genes with the highest mutation rates in gliomas (**Figures S3D, E**). Although mutations in IDH1, TP53 and ATRX were the most frequent in both groups, their mutation frequencies in the PDCL3 low group were higher than those in the PDCL3 high group, which also illustrated, at least in part, that PDCL3 may promote malignant progression and lead to poor prognosis in glioma patients.

### Functional analysis of PDCL3 in glioma

**Figure 4:**
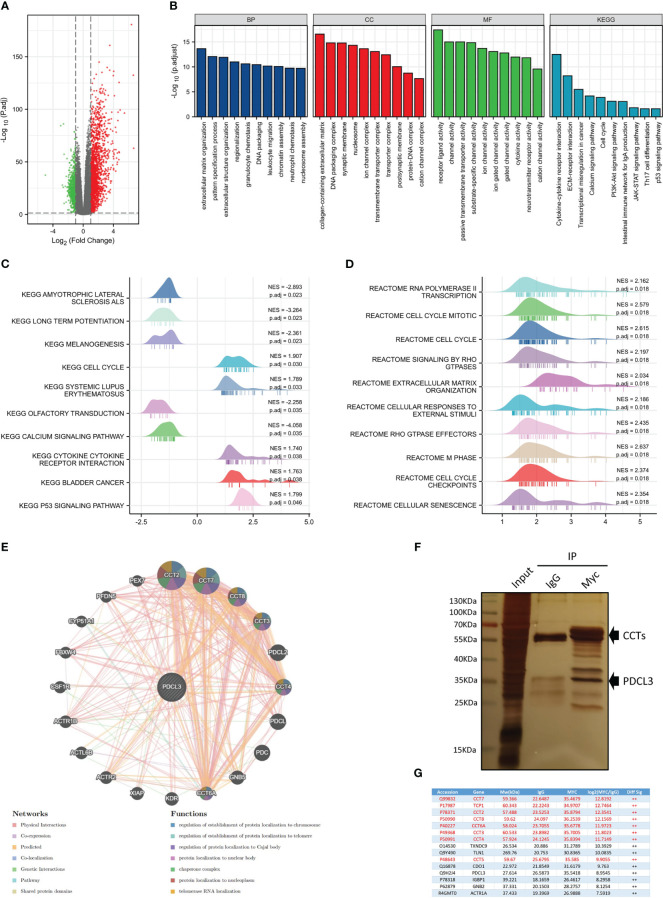
Functional analysis of PDCL3 in glioma. **(A)** Volcano plot showing DEGs between the PDCL3 high and low groups in the TCGA-GBMLGG dataset. **(B)** Histogram showing the top 10 significant terms of BP, MF, CC and KEGG enrichment analysis. **(C, D)** Ridge plot showing the top 10 pathways of GSEA enrichment analysis, including KEGG and Reactome pathways. **(E)** The protein-protein interaction network of PROS1 was constructed using GeneMANIA. **(F)** The immunoprecipitation results were observed using silver staining. **(G)** Mass spectrometry identified the top 15 differentially expressed proteins between the PDCL3-IP group and the IgG-IP group.

To further explore the function of PDCL3 in glioma, PDCL3 high and low groups from RNASeq-Counts of TCGA-GBMLGG were analyzed using the R package “DESeq2”. A total of 2,446 DEGs showed statistically significant group differences, including 1,688 upregulated genes and 758 downregulated genes (**Figure 4A**). Then, we performed functional enrichment analyses to characterize the biological functions of these DEGs between the two groups and depicted a histogram to show the top 10 significant terms of biological process (BP), molecular function (MF) and cell component (CC) in GO terms and the top 10 tumor-related signaling pathways in KEGG enrichment analyses (**Figure 4B**). The enrichment results suggested that PDCL3 may affect not only the malignant biological behavior of tumor cells (cell cycle, transcriptional misregulation in cancer, PI3K-Akt signaling pathway, JAK-STAT signaling pathway, calcium signaling pathway and p53 signaling pathway, etc.) but are also involved in the regulation of the tumor microenvironment (extracellular matrix organization, receptor ligand activity, cytokine activity, ion channel activity, neurotransmitter receptor activity, cation channel activity and substrate-specific channel activity, etc.), especially the immune microenvironment (leukocyte migration and Th17 cell differentiation). Moreover, GSEA, including KEGG (**Figure 4C**) and Reactome (**Figure 4D**) pathways, showed significant enrichment in a series of signaling pathways involved in the regulation of glioma malignant progression and the tumor microenvironment, including the cell cycle, extracellular matrix organization, cytokine-cytokine receptor interaction and p53 signaling pathways. Notably, similar results emerged from the enrichment analysis of the proteome dataset of the GBM cohort in CPTAP. **Figure S4A, B** show the top 50 positively and negatively correlated DEGs with PDCL3. As expected, the GO analyses (**Figure S4C**) revealed significant enrichment in metabolic process, cell communication, cell proliferation, extracellular matrix, protein binding and ion binding. In addition, several cancer-related signaling pathways, including the cell cycle, DNA replication and calcium signaling pathways, were also obtained through GSEA (**Figure S4D**). Above all, functional enrichment analyses suggested that PDCL3 may be involved in regulating the malignant biological process of glioma cells and even the “cross-talk” between tumor cells and other stromal cells in the glioma microenvironment. Furthermore, we predicted the protein-protein interaction network of PDCL3 and identified altered neighboring genes using the GeneMANIA database. In addition to all members of the chaperonin-containing TCP1 complex, PDCL3 may interact closely with kinase insert domain receptor (KDR), colony stimulating factor 1 receptor (CSF1R) and several actin-related proteins (ARPs). (**Figure 4E**). Further validation in the U251 cell line was performed using IP-MS (**Figure 4F**), and the top 15 differentially expressed proteins are shown in **Figure 4G**. Similar results suggested that PDCL3 may achieve its functions by interacting with CCTs in glioma cells.

### Correlation of PDCL3 with immune cell infiltration in the glioma microenvironment

**Figure 5:**
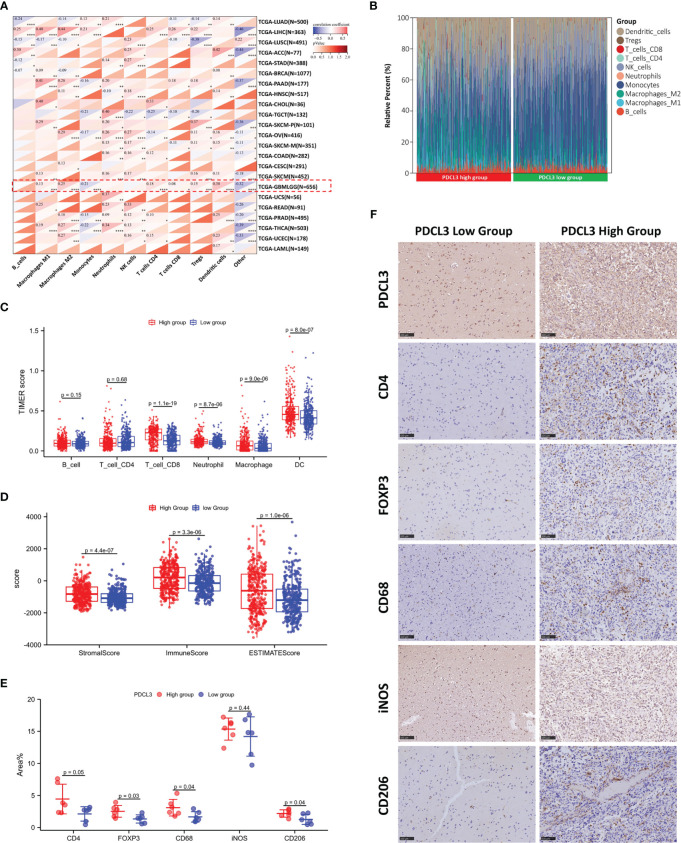
Correlation of PDCL3 with immune cell infiltration in the glioma microenvironment. **(A)** The relationship between PDCL3 expression and immune cell infiltration across cancers was evaluated using the quanTIseq algorithm (*p<0.05, **p<0.01, ***p<0.001, and ****p<0.0001; glioma cohort is shown in red squares). **(B)** The proportion of 10 immune cells in the PDCL3 high and low subgroups. **(C)** Differences in immune cell infiltration between the PDCL3 high and low subgroups in glioma were verified using TIMER. **(D)** Comparison of the immune score, stromal score and ESTIMATE score between the PDCL3 high and low subgroups. **(E)** The expression of 5 immune cell markers was detected in 12 glioma clinical specimens using IHC. The percentage of positive area was used to evaluate the degree of immune cell infiltration. **(F)** Representative IHC staining of five immune cell markers in the PDCL3 high and low groups (20X, scale bars = 100 µm).

Due to the functional enrichment of PDCL3, which is frequently involved in cell-to-cell interactions and immune-related functions, we further investigated the correlation of PDCL3 with the glioma immune landscape. The relationship between the expression of PDCL3 and the infiltration of immune cells across cancers was evaluated using the quanTIseq algorithm, and the results are shown in **Figure 5A**. PDCL3 was positively correlated with the infiltration of multiple immune cells across cancers. Especially in gliomas, PDCL3 expression revealed a positive correlation with M1 macrophages, M2 macrophages, CD4+ T cells, CD8+ T cells, Tregs, and dendritic cell infiltration. The proportions of immune cells in the PDCL3 high and low subgroups are displayed in **Figure 5B**. Meanwhile, differences in immune cell infiltration between the PDCL3 high and low subgroups were verified using the TIMER (**Figure 5C**), ssGSEA (**Figure S5A**) and CIBERSORT (**Figure S5B**) algorithms. From the TIMER results, the PDCL3 high subgroup was accompanied by higher infiltration of CD8+ T cells, macrophages and dendritic cells than the PDCL3 low subgroup. Similar results in macrophages could be observed from ssGSEA. Although there was no significant difference between the two subgroups in the proportion of Tregs, the PDCL3 high subgroup was accompanied by more infiltration of T cells and Th2 cells. From CIBERSORT, despite no difference in M2 macrophages and various dendritic cells, a significant difference was observed in the PDCL3 high subgroup, with higher infiltration of activated CD4+ memory T cells, Tregs, M0 macrophages and M1 macrophages. In addition, the PDCL3 high subgroup had significantly higher immune, stromal and ESTIMATE scores (**Figure 5D**), which also indirectly implied the possible influence of high PDCL3 expression on remodeling the immune microenvironment during glioma development. Furthermore, we randomly selected 12 glioma clinical specimens and detected some immune cell markers, including CD4, FOXP3, CD68, iNOS and CD206, to verify our bioinformatics analysis results. The IHC results suggested that the PDCL3 high group had higher expression of CD4, FOXP3, CD68 and CD206 but no association with iNOS (**Figure 5E**). This means that PDCL3 may be involved in the infiltration of T cells and macrophages, especially their immunosuppressive types, in gliomas. Representative pictures of IHC staining are shown in **Figure 5F**.

### Association between PDCL3 expression and immunomodulators and immune checkpoints

**Figure 6:**
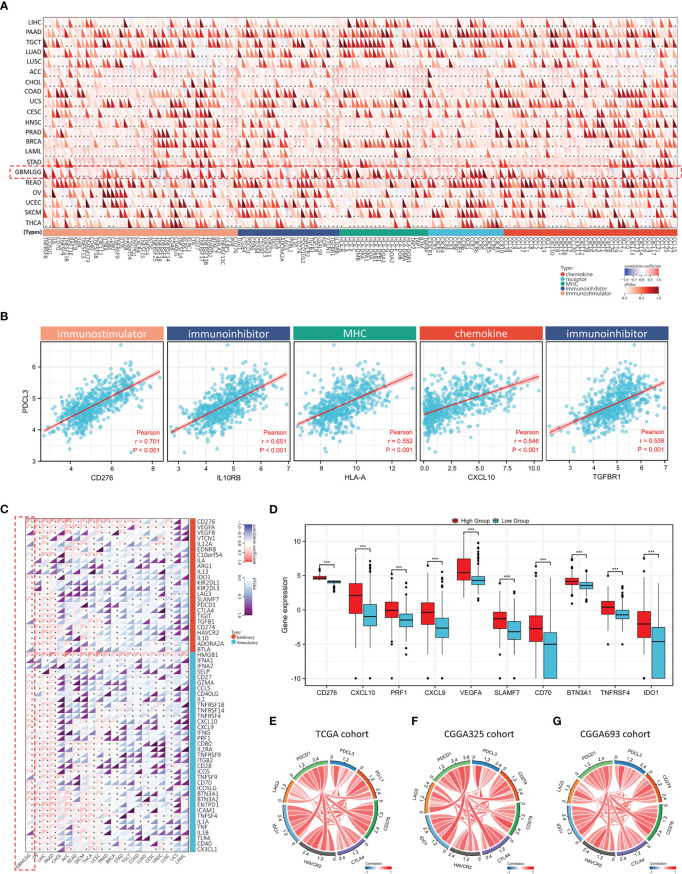
Association between PDCL3 expression and immunomodulators and immune checkpoints. **(A)** Heatmap showing the correlation between PDCL3 expression and 150 common immunomodulators across cancers (*p<0.05; glioma cohort is shown in red squares). **(B)** Scatter diagram showing the correlation between PDCL3 expression and the top 5 relevant immunomodulators. **(C)** Heatmap showing the correlation between PDCL3 expression and 60 common immune checkpoint genes across cancers (*p<0.05; glioma cohort is shown in red squares). **(D)** Box plot showing the expression distribution of the top 10 relevant immune checkpoint genes (***p<0.001). **(E–G)** The correlation between PDCL3 and the 7 most important known immune checkpoints was visualized using chord plots in three independent datasets.

To better understand the interaction between PDCL3 and immune responses, we also explored the association of PDCL3 expression with immunomodulators and immune checkpoints. As shown in **Figure 6A**, PDCL3 expression was positively correlated with most of the 150 common immunomodulators across cancers. Then, we selected the top 5 relevant immunomodulators, including an immunostimulator (CD276), two immunoinhibitors (IL10RB and TGFBR1), a chemokine (CXCL10) and an MHC (HLA-A), in the glioma dataset and drew scatterplots (**Figure 6B**) to show their details. **Figure 6C** illustrates the correlation between PDCL3 and 60 common immune checkpoint genes (24 inhibitory and 36 stimulatory) across cancers. Notably, most of them (18 inhibitory and 31 stimulatory) showed significant correlations in the glioma dataset. **Figure 6D** shows the expression distribution of the top 10 relevant immune checkpoint genes (CD276, CXCL10, PRF1, CXCL9, VEGFA, SLAMF7, CD70, BTN3A1, TNFRSF4 and IDO1) in the PDCL3 high and low subgroups. The association between PDCL3 and the 7 most important known immune checkpoints (PD-1, PD-L1, CTLA4, LAG3, HAVCR2, CD276 and IDO1) was visualized using chord plots. The consistent results obtained from three independent datasets indicated the stability and reliability of the conclusion (**Figures 6E–G**). Taken together, these results strongly imply that PDCL3 may play a critical role in glioma immunoregulation.

### PDCL3 is associated with cancer stemness and angiogenesis

**Figure 7:**
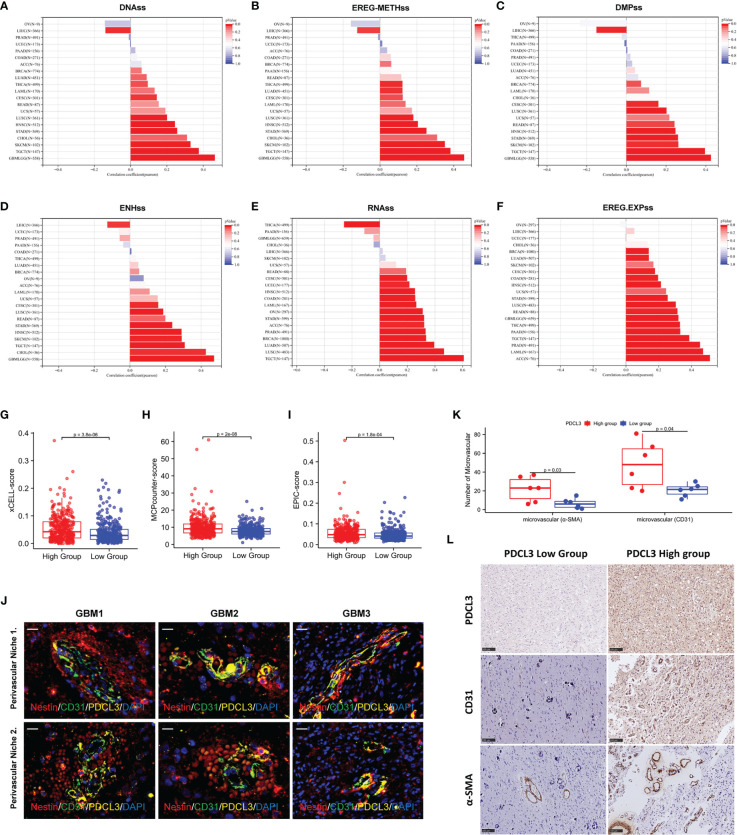
PDCL3 is associated with cancer stemness and angiogenesis. **(A–F)** Six different stemness indexes, including DNAss, EREG-METHss, DMPss, ENHss, RNAss and EREG.EXPss were calculated with PDCL3 expression in pan-cancer. **(G–I)** Three different algorithms, including xCELL, MCPcounter and EPIC, were used to evaluate the infiltration of endothelial cells in the PDCL3 high and low subgroups. **(J)** Immunofluorescence showed the distribution relationship of PDCL3, CD31 and Nestin in 3 GBM clinical specimens (60X, scale bars = 50 µm). **(K)** The expression of CD31 and α-SMA was detected in 12 glioma clinical specimens using IHC. The microvasculature per field was used to evaluate the relationship between PDCL3 expression and angiogenesis. **(L)** Representative IHC staining of CD31 and α-SMA in the PDCL3 high and low groups (20X, scale bars = 100 µm).

Glioma stem cells and angiogenesis, as independent factors in the remodeling of the glioma immune microenvironment, were also explored for their relationship with PDCL3 expression. We first analyzed the relationship between PDCL3 expression and cancer stemness across cancers by calculating six different stemness indexes. The results indicated that DNA methylation-based stemness (DNAss, **Figure 7A**), epigenetically regulated DNA methylation-based stemness (EREG-METHss, **Figure 7B**), differentially methylated probe-based stemness (DMPss, **Figure 7C**), enhancer element/DNA methylation-based stemness (ENHss, **Figure 7D**), RNA expression-based stemness (RNAss,**Figure 7E**) and epigenetically regulated RNA expression-based stemness (EREG.EXPss, **Figure 7F**) were positively correlated with PDCL3 expression in most types of cancers, especially gliomas (DNAss: R=0.47, P=1.09e-31; EREG-METHss: R=0.46, P=5.34e-30; DMPss: R=0.43, P=6.35e-26; ENHss: R=0.47, P=2.26e-32; EREG.EXPss: R=0.32, P=3.87e-17). Next, we explored the association between PDCL3 expression and the infiltration of endothelial cells using three different algorithms. Consistent results from xCELL, MCPcounter and EPIC showed that the degree of endothelial cell infiltration in the PDCL3 high group was significantly higher than that in the low group, which suggested that gliomas with high PDCL3 were often accompanied by more angiogenesis (**Figures 7G–I**). Interestingly, we detected three GBM samples using immunofluorescence triple staining and observed a segment of PDCL3-positive expression in the “oversleeve-like” region formed by Nestin and CD31 (**Figure 7J**). This implies that PDCL3 may also be involved in the regulation of glioma stem cell perivascular niches. Moreover, we also detected microvascular numbers of the abovementioned 12 glioma specimens by IHC staining for CD31 and α-SMA. The results were consistent with those of bioinformatics analysis (**Figure 7K**). Representative pictures of IHC staining are shown in **Figure 7L**.

### Experimental validation of PDCL3 function in glioma cell lines

**Figure 8:**
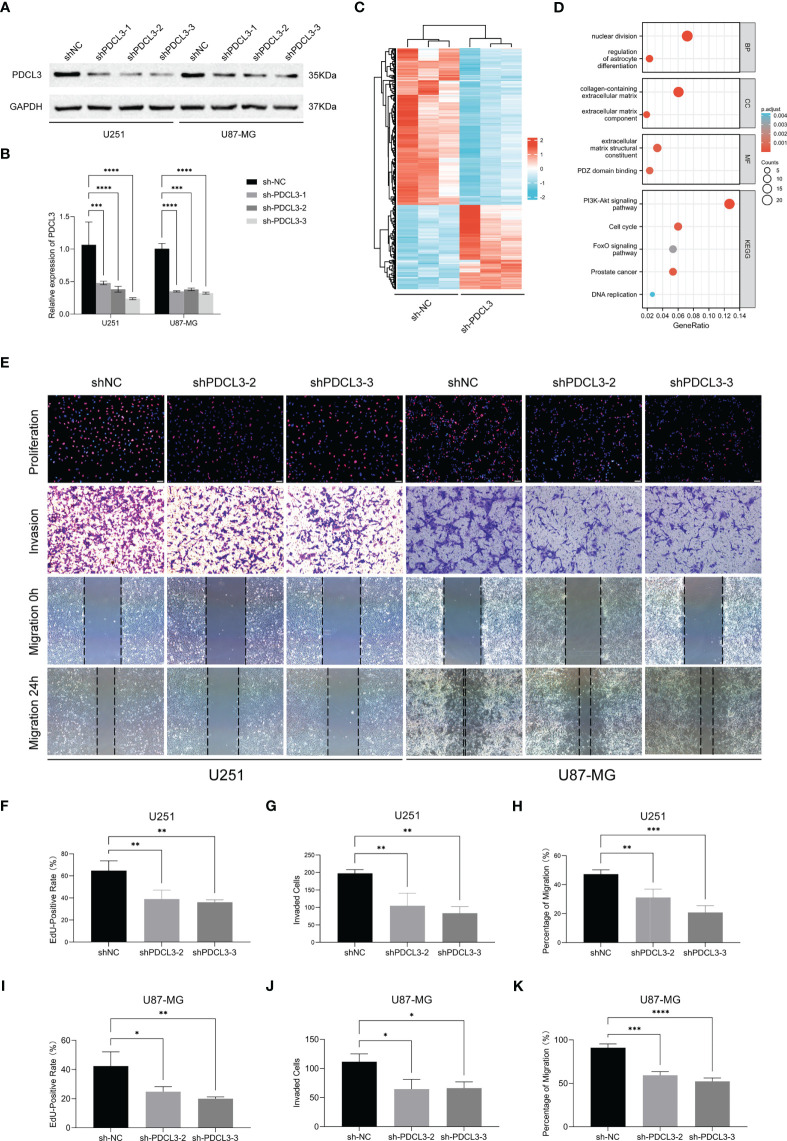
Experimental validation of PDCL3 function in glioma cell lines. **(A, B)** PDCL3 interference efficiency was detected using Western blotting and qRT-PCR (**p<0.01). **(C)** Heatmap revealing the 327 DEGs identified by transcriptome sequencing analysis. **(D)** Bubble diagram showing the results of GO and KEGG pathway enrichment. **(E)** Representative pictures of EdU, transwell and wound healing assays in U251 and U87-MG cells. **(F–K)** Statistical results of EdU, transwell and wound healing assays in U251 and U87-MG(*p<0.05, **p<0.01, ***p<0.001).

To clarify the function of high PDCL3 expression in glioma cells, we performed a series of cell biology experiments. First, we constructed the stable glioma cell lines U251 and U87-MG with low expression of PDCL3 by RNA interference (RNAi) and tested its interference efficiency by Western blotting and qRT-PCR (**Figures 8A, B**). Then, we selected the shNC and shPDCL3-3 groups for transcriptome sequencing analysis (biological replicates three times). As shown in **Figure 8C**, 212 genes were upregulated and 115 genes were downregulated in the shPDCL3 groups compared with the shNC groups. The GO and KEGG pathway enrichment showed that DEGs were associated with malignant progression of glioma cells, including nuclear division, ECM component, PDZ domain binding, cell cycle, DNA replication, PI3K-Akt and FoxO signaling pathways, which was partially consistent with the results of bioinformatics analysis in the public database (**Figure 8D**). Finally, we detected the proliferation, invasion and migration of glioma cells in a conventional experimental manner (**Figure 8E**). The results of the EdU assay showed that knockdown of PDCL3 significantly inhibited proliferation in U251 and U87-MG cells (**Figures 8F, I**). Transwell assays suggested that knockdown of PDCL3 could significantly inhibit the invasiveness of U251 and U87-MG cells (**Figures 8G, J**). In addition, a wound healing assay showed that knockdown of PDCL3 significantly inhibited the migration ability of U251 and U87-MG cells (**Figures 8H, K**). In summary, high expression of PDCL3 mediates the malignant progression of glioma cells, and it is a novel oncogene in glioma.

### Verification of PDCL3 function and expression at the single-cell level

**Figure 9:**
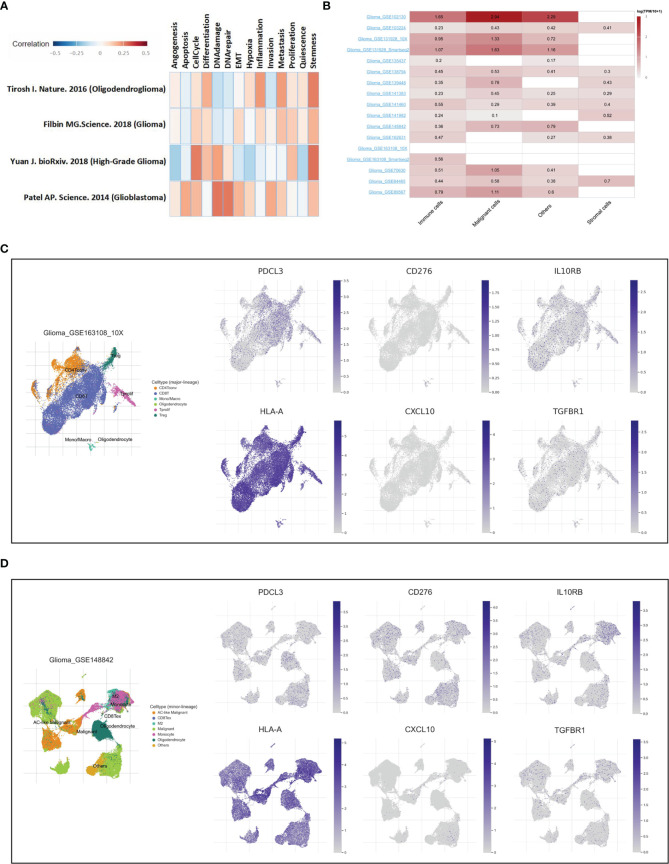
Verification of PDCL3 function and expression at the single-cell level. **(A)** Functional analysis of PDCL3 in glioma using the CancerSEA database. **(B)** The expression of PDCL3 in different cell types from 17 independent single-cell transcriptome sequencing datasets. **(C, D)** Two representative datasets, GSE163108_10X and GSE148842, show the expression and distribution of PDCL3, CD276, IL10RB, HLA-A, CXCL10 and TGFBR1.

Single-cell RNA sequencing is a powerful approach to reveal the cellular and transcriptional heterogeneity of complex tissues. Here, two single-cell sequencing databases, CancerSEA and TISCH, were used to further verify the function and expression of PDCL3 in glioma. As shown in **Figure 9A**, PDCL3 positively influenced the cell cycle, DNA damage and repair, inflammation, cell invasion and metastasis, and cancer stemness. **Figure 9B** shows the expression of PDCL3 in different cell types from 17 independent single-cell transcriptome sequencing datasets. PDCL3 was expressed in various glioma cell lineages, in which immune cells and malignant cells were the most abundant. Representative datasets (GSE163108_10X and GSE148842) are shown in **Figures 9C, D**. In the GSE163108_10X dataset, PDCL3 was mainly expressed in Tregs, Tprolif cells, mono/macro cells, and some CD4+ T cells and CD8+ T cells, and its expression region partly overlapped with IL0RB, HLA-A and TGFBR1. In the GSE148842 dataset, PDCL3 was positively expressed in monocytes, M2 macrophages, oligodendrocytes and various malignant cells, and its expression region also partly overlapped with CD276 and CXCL10. The results of single-cell transcriptome sequencing analysis perfectly supported our previous conclusions and provided a macroscopic landscape of PDCL3 in glioma.

## Discussion

In this study, we reported a novel prognosis-related biomarker, PDCL3, and its pathological role in glioma for the first time. Phosducin was originally found in the retina because of its association with purified transducin Gβγ (21). Although it is best known for its role in the visual system, phosducin has also been proposed to regulate other signaling systems (22). At present, phosducin and several phosducin-like proteins (PhLPs) are known to be expressed in various tissues, including the brain, liver, pineal, and olfactory epithelium (23); however, there are few reports on their relationship with human cancers. This is the first comprehensive evaluation of PDCL3 expression and its related functions and mechanisms possibly underlying carcinogenesis in patients with malignant glioma. At the beginning of this study, we identified that PDCL3 was upregulated in various cancer tissues and strongly correlated with poor prognosis in glioma patients. Then, we performed validation using three glioma public datasets (CGGA325, CGGA693 and CGGA301) and glioma specimen cohorts from two different medical centers (Wuhan Union Hospital and Renmin Hospital of Wuhan University). Consistent results from multiple medical centers, multiple public datasets and multiple different detection methods fully demonstrated the reliability and stability of PDCL3 as a prognosis-related biomarker in glioma. Moreover, we also constructed a prognostic nomogram using PDCL3 expression combined with other conventional clinical indicators for the clinical use of this new biomarker. The satisfactory prognostic efficiency of this nomogram also demonstrated the value of PDCL3 in predicting glioma prognosis.

To better understand the functional effects of upregulated PDCL3 in gliomas, we investigated it from the perspectives of epigenetic modification, gene mutation, gene function and protein function by analyzing mult-omics data. PDCL3 expression is closely related to multiple DNA and RNA modification genes, which implies that PDCL3 may mediate epigenetic modification directly or indirectly despite insufficient and reliable experiments. From functional enrichment analysis of genes and proteins, we found that upregulated PDCL3 may not only maintain the malignant biological behavior of glioma cells but also be involved in remodeling the tumor microenvironment. For the former, we performed a series of cell biology experiments, including RNAi, RNA-seq, EdU, Transwell and wound healing assays, and confirmed this inference. For the latter, we used multiple bioinformatics algorithms combined with immunohistochemical detection of clinical specimens for verification. On the other hand, from studies to date, it appears that PDCL3 may act as a co-chaperone with CCT in protein folding (9, 24). In this study, we used an IP-MS assay to explore proteins interacting with PDCL3 in glioma cells. The results are basically consistent with those of previous studies. Several studies have investigated whether CCT dysfunction is closely related to the mechanisms of carcinogenesis (25). For example, overexpression of CCT2 could promote triple-negative breast cancer cell chemoresistance and cell migration and invasion *via* the AKT/GSK3β/β-catenin and XIAP/Survivin pathways (26). CCT4 may facilitate glioblastoma cell growth by involving the YB-1/CCT4/mLST8/mTOR signaling pathway (27). Upregulated CCT5 could induce epithelial-mesenchymal transition to promote gastric cancer lymph node metastasis by activating the Wnt/β-catenin signaling pathway (28). CCT8 could recover WTp53-suppressed cell cycle evolution and EMT to promote colorectal cancer progression (29). However, the relationship between PDCL3 imbalance and CCT dysfunction in carcinogenesis needs further experimental verification.

It is well known that gliomas are “cold immune” tumors (30). Compared with other types of solid tumors, the immunosuppressive properties of gliomas are more intense, which partly leads to the lack of effectiveness of various passive immunotherapies in gliomas (31, 32). Although the proportion of immune cell infiltration in gliomas is relatively low and some GBMs even present the characteristics of an “immune desert”, this does not indicate the insignificance of infiltrating immune cells in the glioma microenvironment (33). In contrast, these infiltrated immune cells are acclimated by the special microenvironment, assisting tumor cell immune escape, promoting malignant progression, and finally leading to the failure of glioma therapy (34). Recent studies have shown that high infiltration of microglia/macrophages is a characteristic of the glioma microenvironment (35). The M2 subtype, which has immunosuppressive properties, is more prevalent in high-grade gliomas than in low-grade gliomas (36). Significantly, M2-type macrophages have multiple effects on the glioma microenvironment, including increased angiogenesis, remodeled extracellular matrix, and enhanced tumor cell invasion (37). Thus, elevated M2-type macrophage numbers are associated with poor prognosis in gliomas. Another immunosuppressive cell type that has been shown to be present in gliomas is Tregs (38). Several studies have suggested that the proportion of Tregs increases with the grade of gliomas, and a higher proportion of Tregs also predicts shorter survival (39, 40). Tregs significantly inhibit the activation of effector T cells by binding CTLA-4 to CD80 or CD86 (41). In addition, FoxP3+ Tregs can directly inhibit T-cell proliferation by inducing HO-1 expression (42). Moreover, Tregs also inhibit the secretion of proinflammatory factors (such as IL-2 and IFN-γ, etc.) and facilitate the secretion of immunosuppressive factors (such as TGF-β and IDO, etc.) (43), which caused the functional inhibition of dendritic cells, antigen presenting cells and other lymphocytes, as well as more severe immunosuppression (44). In this study, the results of various bioinformatic algorithms and immunohistochemical experiments in glioma clinical specimens showed more infiltration of M2-type macrophages and Tregs in the PDCL3 high group, which suggested that high expression of PDCL3 was positively correlated with characteristics of immunosuppression in gliomas. On the other hand, PDCL3 expression was also associated with various immune checkpoints and immunomodulators. In particular, its high correlation with IL-10RB and TGFRB suggested that PDCL3 may be involved in the immunosuppression of glioma. Notably, the most relevant one, CD276 (B7H3), is a potential target of CAR-T products, which exhibit promising efficacy in the treatment of glioblastoma both *in vitro* and *in vivo (* 45). This means that PDCL3 could potentially be a predictor of the efficacy of CAR-T therapy. Although there is insufficient experimental evidence to prove how PDCL3 directly regulates the immune landscape, our results confirmed the high correlation between PDCL3 expression and glioma immunosuppression. This also explains why patients with high levels of PDCL3 have poor prognosis from the perspective of the glioma immune microenvironment.

Although the glioma microenvironment contains many other components, it is undeniable that abundant microvascular infiltration is one of the best features. Because of aberrant vasculature with vessels of variable diameter, heterogeneous distribution, and increased blood-brain barrier (BBB) permeability, this defective neovascularization not only leads to malignant edema, tissue hypoxic necrosis and other pathological changes but also provides a pathological basis for immune cell infiltration and the formation of a special immunosuppressive microenvironment in gliomas (46–48). Neovascularization in gliomas is of high clinical relevance, as it correlates with biological aggressiveness, degree of malignancy, clinical recurrence, and postoperative survival (46). On the other hand, glioma stem cells (GSCs), as a special presence in the tumor microenvironment, also make great contributions to angiogenesis and immunoregulation (49). Recent studies have shown that GSCs are often located in perivascular niches and interact with ECs in a bidirectional manner (50). GSCs can not only directly secrete a variety of vascular stimulating factors but also be recruited to endothelial cells through the SDF-1/CXCR4 axis and differentiate into pericytes under TGF-β induction (51). GSCs also induce the M2 phenotype in macrophages, suppressing an immune response and enabling tumor evasion from immune cell clearance (52). Moreover, GSCs promote the recruitment of myeloid cells through epigenetic immune editing (rather than subclonal selection), forming a myeloid cell-rich tumor microenvironment and achieving immune evasion and tumor progression (53). In this study, we demonstrated that PDCL3 expression was significantly correlated with glioma angiogenesis and cancer stemness. In addition, PDCL3 was also positively expressed in the prevascular niche of GSCs. These findings may also imply that PDCL3 could contribute to the construction of the prevascular niche and angiogenesis. Significantly, PDCL3 may act as a chaperone protein that increases the stability of VEGFR-2 by inhibiting its ubiquitination (12). This mechanism enables the knockdown of PDCL3 to antagonize the effect of VEGFA on endothelial cells and inhibit angiogenesis. Focusing on the role of PDCL3 in the glioma vascular microenvironment, our study complements previous studies. As a focal point involved in the vascular microenvironment and the immune microenvironment, the regulatory mechanism of PDCL3 in the glioma microenvironment deserves further exploration in the future.

Due to the limitations of experimental conditions and research funds, our research still has some deficiencies. First, we did not design an ideal patient-derived tumor xenograft (PDX) model to verify the effects of PDCL3 *in vivo*. Second, the effect of PDCL3 on glioma immunosuppression and the anti-PD-1 immunotherapy response still needs to be further verified *in vitro* and *in vivo*. Finally, although this study aims to reveal PDCL3 as a novel prognostic marker of glioma from multiple perspectives, more mechanisms and regulatory details should be explored in depth.

## Conclusion

In summary, this study elucidated that PDCL3 is upregulated in various types of human cancers and may serve as a novel prognostic biomarker in gliomas. The association of PDCL3 with the infiltration of immune cells, immunomodulatory genes, immune checkpoints, cancer stemness and angiogenesis suggested that PDCL3 may be involved in regulating the immune landscape of the glioma microenvironment. Knockdown of PDCL3 decreased the proliferation, invasion and migration of glioma cells, which impled the oncogenic role of PDCL3. This function of PDCL3 may lead to poor prognosis of gliomas. Mechanistically, PDCL3 may participate in the regulation of metabolic processes, cell communication, cell proliferation and the extracellular matrix by binding CCT and affecting the folding of some important proteins. Our study provides a novel biomarker with value in assisting clinical diagnosis, predicting patient outcomes and assessing the immune landscape of the tumor microenvironment in glioma.

## Data availability statement

The data presented in the study are deposited in the Open Archive for Miscellaneous Data (OMIX) repository, accession number: OMIX003261 http://ngdc.cncb.ac.cn/omix/view/omix003261.

## Ethics statement

The studies involving human participants were reviewed and approved by Institutional Ethics Committee of Tongji Medical College, Huazhong University of Science and Technology. The patients/participants provided their written informed consent to participate in this study.

## Author contributions

ZP, DY, and WX conceived and designed this study. ZP performed the bioinformatics analysis. JW performed qRT-PCR detection. ST and YW performed IHC and WB detection. ZP and DY performed other cell biology experiments. ZP performed the data analysis, figure plotting and writing. WX and DY were responsible for the critical reading of the manuscript. WX revised the manuscript. All authors contributed to the article and approved the submitted version.

## Supplementary material

Supplementary Table 1 The clinicopathological characteristics of 94 patients in our cohort.

Supplementary Table 2 The clinicopathological characteristics of patients in the glioma tissue microarray.

Supplementary Table 3 Details of all primer sequences

Supplementary Table 4 List of antibody information

Supplementary Table 5 PDCL3 expression association with clinical pathological characteristics (logistic regression)

Supplementary Figure 1 PDCL3 expression in multiple human cancer cell lines. **(A)** Data from the CCLE database. **(B)** Data from the HPA database.

Supplementary Figure 2 The ROC curves quantified the diagnostic efficacy and prognostic power of PDCL3. **(A–D)** ROC curve analysis confirmed the diagnostic accuracy of PDCL3 in the four cohorts. **(E–H)** Time-dependent ROC curve analysis confirmed a satisfactory prediction performance of PDCL3 in the four cohorts.

Supplementary Figure 3 PDCL3 expression is associated with epigenetic modifications and genetic mutations. **(A)** PDCL3 expression is closely related to the expression of 44 RNA modification genes across cancers. **(B)** PDCL3 expression is closely related to the expression of 4 DNA methyltransferase genes across cancers. **(C)** Lollipop chart showing the PDCL3 mutation distribution and types in its protein domains. **(D, E)** Waterfall diagrams presenting the top 15 genes with the highest mutation rates in the high and low PDCL3 groups of gliomas.

Supplementary Figure 4 LinkOmics was applied to online analyze the proteome dataset of the GBM cohort in CPTAP. **(A, B)** Heatmaps show the significantly positively and negatively correlated genes with PDCL3. **(C, D)** Functional enrichment analysis was performed using GO terms and GSEA.

Supplementary Figure 5 Immune cell infiltration was evaluated using ssGSEA **(A)** and CIBERSORT **(B)**.

## Glossary

**Table:**

GBM	glioblastoma multiforme
LGG	lower-grade glioma
IDH	isocitrate dehydrogenase
MGMTp	O 6 -methylguanine-DNA methyltransferase promoter
TCGA	The Cancer Genome Atlas Project
PDCL3	Phosducin-like 3
PhLP2A	Phosducin-like Protein 2A
VIAF	Viral IAP associated factor
CCT	chaperone proteins containing TCP-1
PhLP3	Phosducin-like Protein 3
TXNDC9	Thioredoxin domain-containing 9
CGGA	Chinese Glioma Genome Atlas
GTEx	Genotype-Tissue Expression database
CCLE	Cancer Cell Line Encyclopedia database
FPKM	fragments per kilobase of exon model per million mapped reads
TPM	Transcripts Per Kilobase of exon model per Million mapped reads
KM	Kaplan‒Meier
ROC curve	receiver operating characteristic curve
DEGs	differential genes
GO	Gene Ontology
KEGG	Kyoto Encyclopedia of genes and Genomes
GSEA	gene set enrichment analysis
CPTAP	clinical proteomic tumor analysis consortium
DMEM	Dulbecco’s modified Eagle’s medium
qRT‒PCR	Quantitative Real-Time Polymerase Chain Reaction
IP-MS	immunoprecipitation-mass spectrometry
IF	immunofluorescence
IHC	immunohistochemistry
DAB	diaminobenzidine
AOD	average optical density
IOD	integral optical density
EdU	5-Ethynyl-2’-deoxyuridine
BRCA	breast invasive carcinoma
CHOL	cholangiocarcinoma
COAD	colon adenocarcinoma
ESCA	esophageal carcinoma
HNSC	head and neck squamous cell carcinoma
KICH	kidney Chromophobe
LIHC	liver hepatocellular carcinoma
LUAD	lung adenocarcinoma
LUSC	lung squamous cell carcinoma
PRAD	prostate adenocarcinoma
STAD	stomach adenocarcinoma
UCEC	uterine corpus endometrial carcinoma
CESC	cervical squamous cell carcinoma and endocervical adenocarcinoma
SKCM	skin cutaneous melanoma
THCA	thyroid carcinoma
READ	rectum adenocarcinoma
OV	ovarian serous cystadenocarcinoma
PAAD	pancreatic adenocarcinoma
TGCT	testicular germ cell tumors
UCS	uterine carcinosarcoma
ALL	acute lymphoblastic leukemia
LAML	acute myeloid leukemia
ACC	adrenocortical carcinoma
OS	overall survival
PFS	progression-free survival
AUC	area under curve
m1A	N1-methyladenosine
m5C	cytosine-5-methylation
m6A	N6-methyladenosine
SNV	simple nucleotide variation
BP	biological process
MF	molecular function
CC	cell component
DNAss	DNA Methylation-based stemness
EREG-METHss	DNA Methylation-based stemness
ENHss	Enhancer Elements/DNAmethylation-based stemness
DMPss	Differentially Methylated Probes-based stemness
RNAss	RNA Expression-based stemness
EREG. EXPss	Epigenetically Regulated RNA Expression-based stemness
RNAi	RNA interference
TISCH	Tumor Immune Single-cell Hub
GSCs	glioma stem cells.
