# Supplementary figures and images for: Phosducin-like 3 is a novel prognostic and onco-immunological biomarker in glioma: A multi-omics analysis with experimental verification
Source: Front Immunol. 2023 Mar 15;14:1128151. doi: 10.3389/fimmu.2023.1128151 (PMC10050339; doi:10.3389/fimmu.2023.1128151)

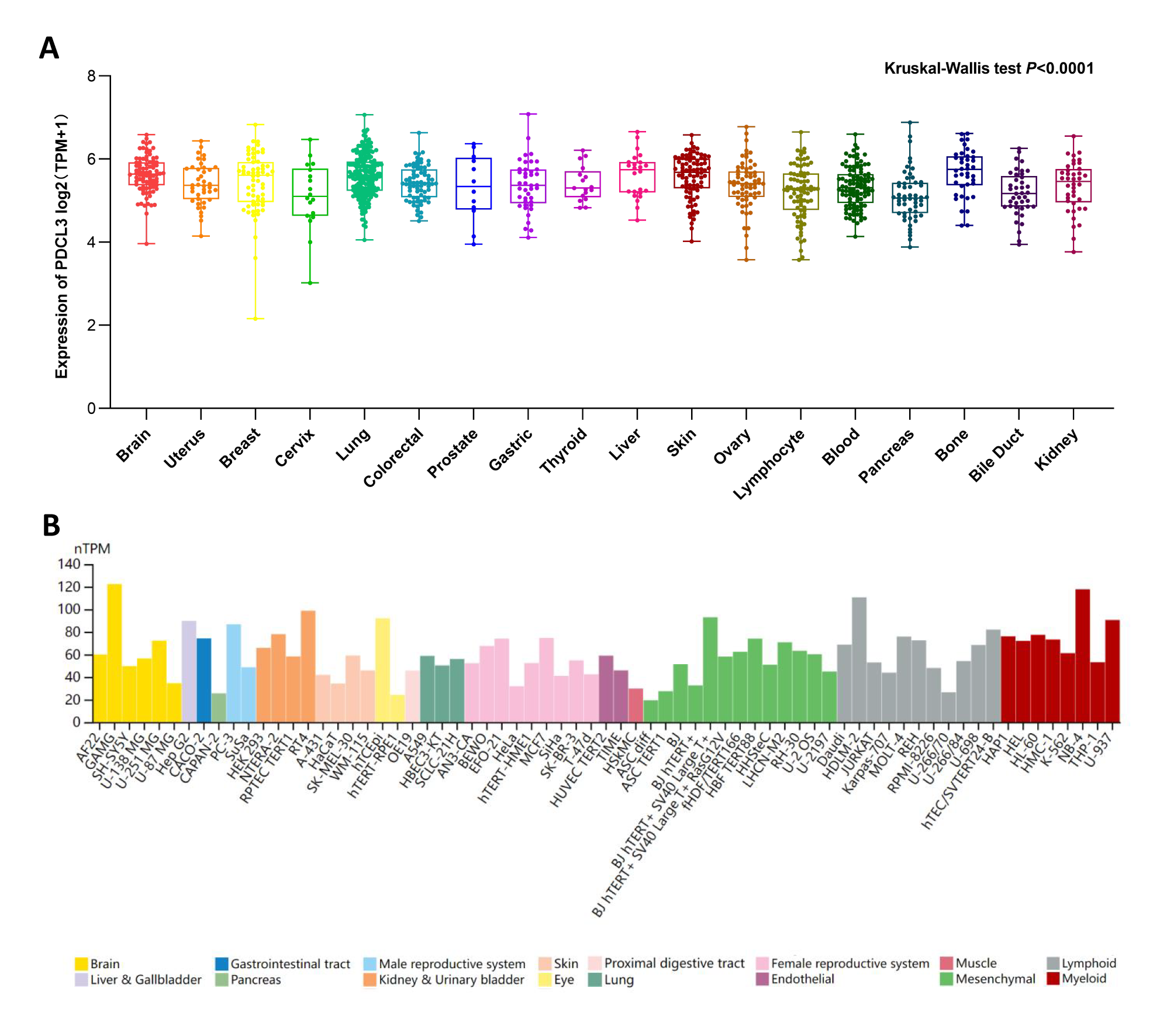

Supplement: Supplementary Figure 1 — PDCL3 expression in multiple human cancer cell lines. (A) Data from the CCLE database. (B) Data from the HPA database. [file Image_1.tif]

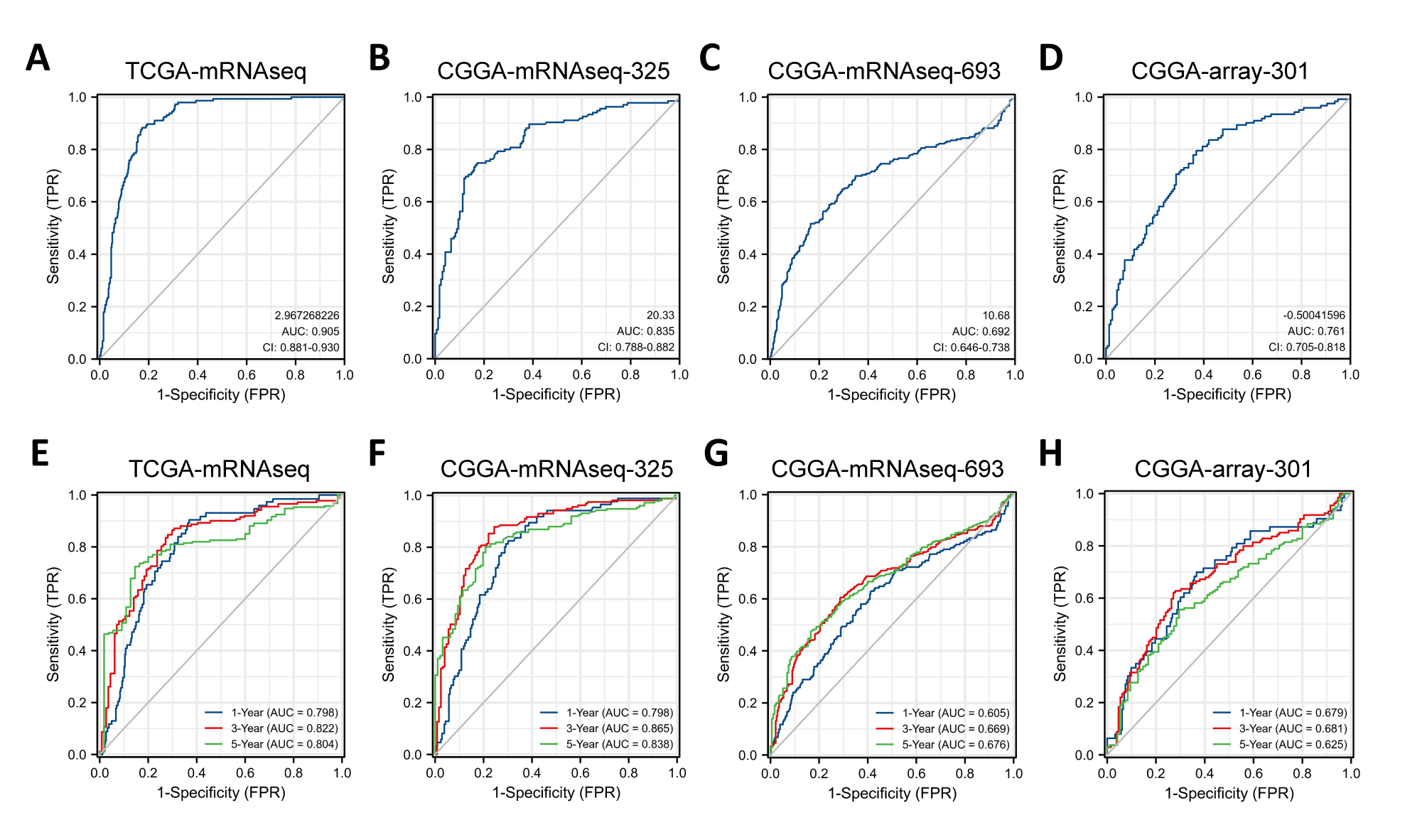

Supplement: Supplementary Figure 2 — The ROC curves quantified the diagnostic efficacy and prognostic power of PDCL3. (A–D) ROC curve analysis confirmed the diagnostic accuracy of PDCL3 in the four cohorts. (E–H) Time-dependent ROC curve analysis confirmed a satisfactory prediction performance of PDCL3 in the four cohorts. [file Image_2.tif]

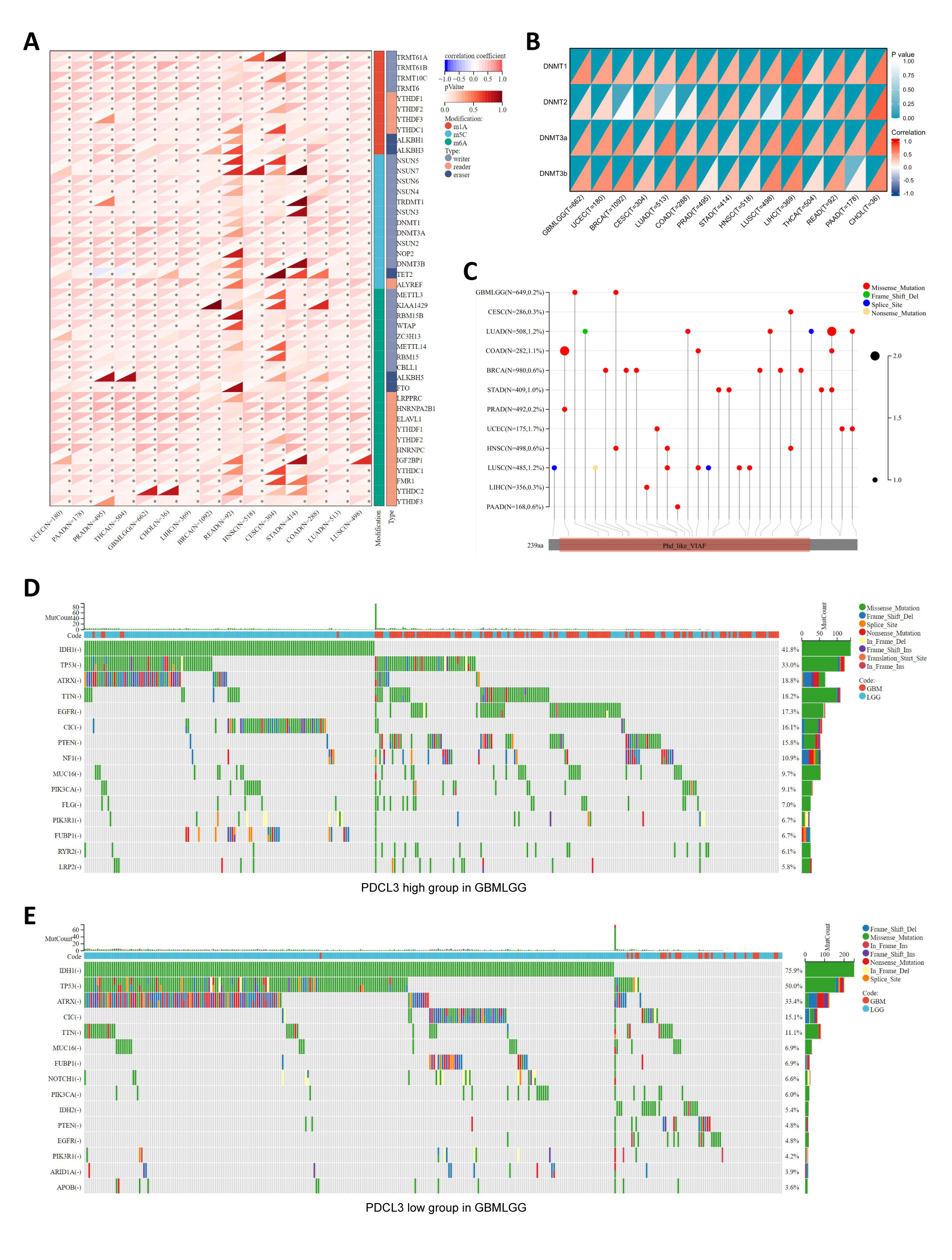

Supplement: Supplementary Figure 3 — PDCL3 expression is associated with epigenetic modifications and genetic mutations. (A) PDCL3 expression is closely related to the expression of 44 RNA modification genes across cancers. (B) PDCL3 expression is closely related to the expression of 4 DNA methyltransferase genes across cancers. (C) Lollipop chart showing the PDCL3 mutation distribution and types in its protein domains. (D, E) Waterfall diagrams presenting the top 15 genes with the highest mutation rates in the high and low PDCL3 groups of gliomas. [file Image_3.tif]

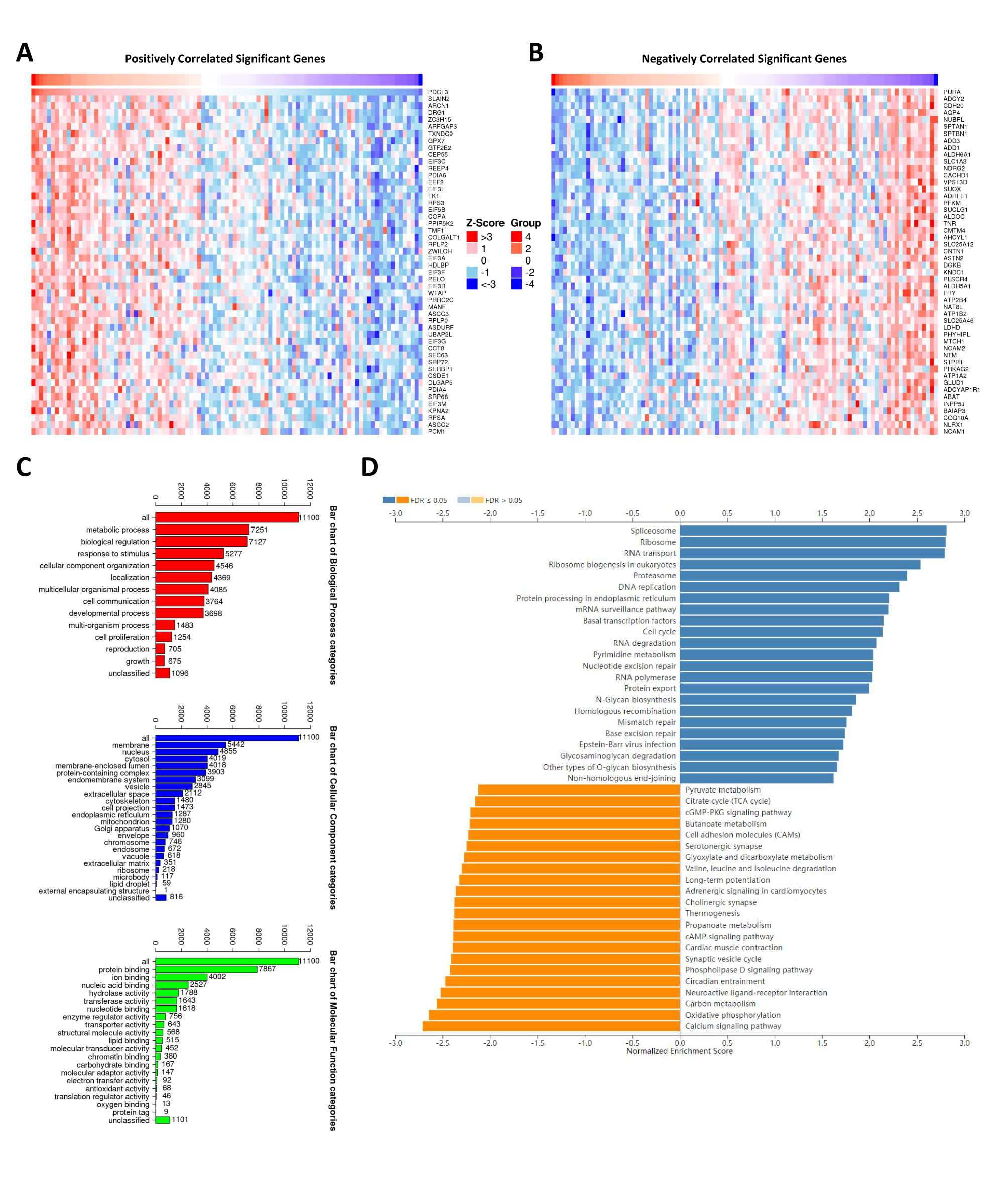

Supplement: Supplementary Figure 4 — LinkOmics was applied to online analyze the proteome dataset of the GBM cohort in CPTAP. (A, B) Heatmaps show the significantly positively and negatively correlated genes with PDCL3. (C, D) Functional enrichment analysis was performed using GO terms and GSEA. [file Image_4.tif]

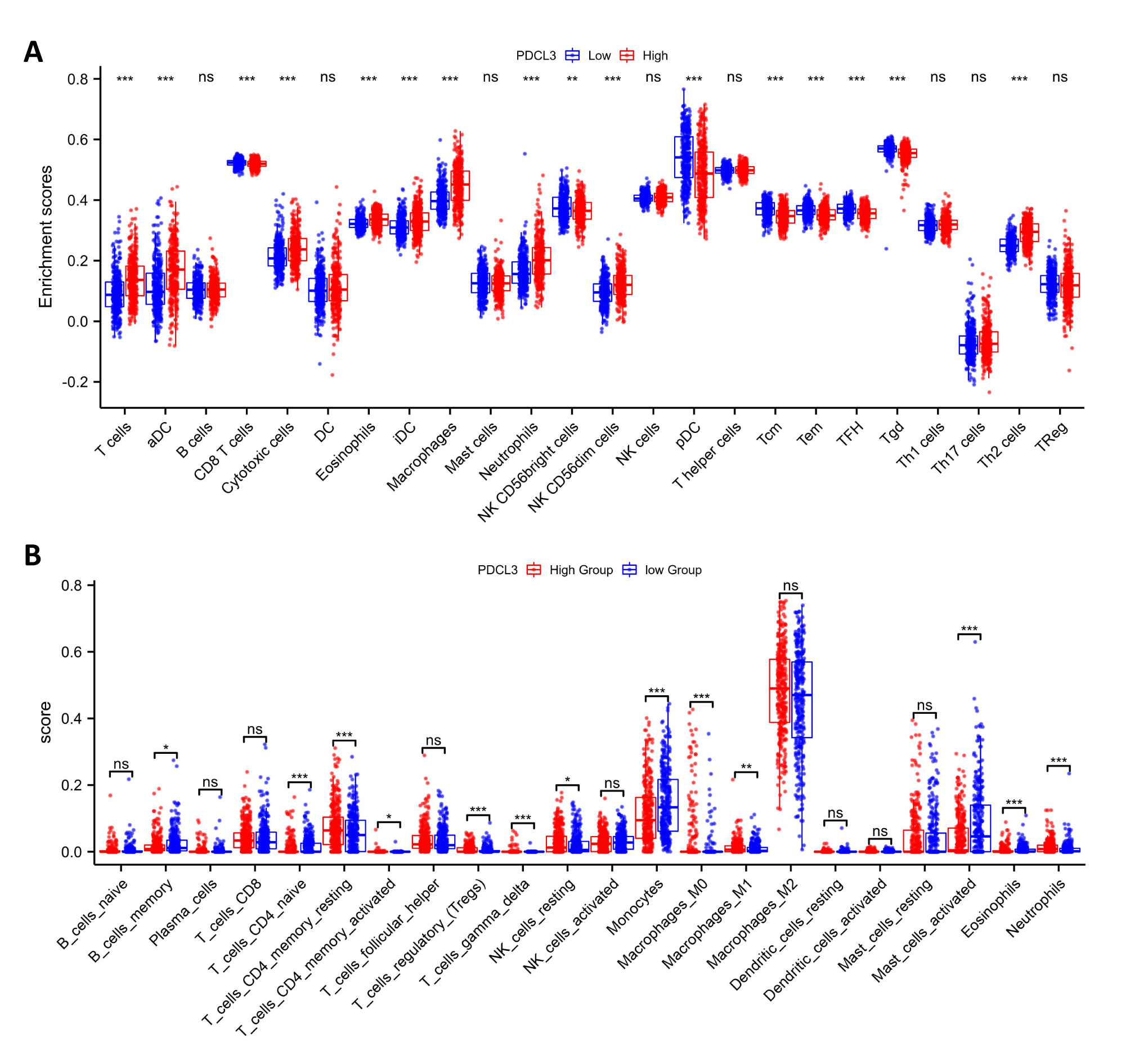

Supplement: Supplementary Figure 5 — Immune cell infiltration was evaluated using ssGSEA (A) and CIBERSORT (B). [file Image_5.tif]
